# Construction and validation of a high-precision annotated dataset for developing intelligent critical vein recognition models in laparoscopic pancreatic surgery

**DOI:** 10.3389/fsurg.2026.1711392

**Published:** 2026-02-02

**Authors:** Hu Zhou, Lu Ping, Ruohan Cui, Junyi Gao, Xianlin Han, Wenming Wu, Surong Hua

**Affiliations:** 1 Department of General Surgery, State Key Laboratory of Complex, Severe, and Rare Diseases, Peking Union Medical College Hospital, Chinese Academy of Medical Sciences & Peking Union Medical College, Beijing, China; 2 Chinese Academy of Medical Sciences & Peking Union Medical College, Beijing, China; 3 Beijing United Family Hospital, Beijing, China

**Keywords:** benchmark metrics, dataset, deep learning, laparoscopic pancreatic surgery, semantic segmentation, vein recognition

## Abstract

**Background:**

Laparoscopic operation holds multiple advantages as a minimal invasive method of surgical treatment. Vascular-related manipulations, including identification and dissection of vascular structures and control of bleeding, are highly experience-based and demand a tortuous learning curve. With the rapid development of artificial intelligence (AI) in the entire diagnosis and treatment process of diseases, data-driven AI models have shown promising potentials in both education and real-time aiding in surgery. However, there is no dedicated dataset existing for developing vascular identification models in laparoscopic settings.

**Methods:**

Videos from 23 laparoscopic distal pancreatectomy (LDP) and laparoscopic pancreaticoduodenectomy (LPD) performed at Peking Union Medical College Hospital (PUMCH) between January 2021 and June 2022 were collected. Senior surgeons systematically reviewed surgical videos to visually identify critical venous vasculature, precisely annotating frame-accurate start and end timestamps on the video timeline. Frames were extracted from these video segments at a fixed temporal interval of one frame per second, then stored to compile the source image dataset. The contours of superior mesenteric vein (SMV), portal vein (PV), splenic vein (SV) were labelled and reviewed according to standard procedure. A High-Resolution Network (HRNet) was combined with a fully convolutional network (FCN) output head to construct a preliminary segmentation model for initial validation and comparison.

**Results:**

A dataset comprises 19,003 annotated frames and is publicly available. The baseline model achieved a recall of 79.6%, precision of 95.8%, and Dice coefficient of 0.69 on the testing set.

**Conclusion:**

This study constructed and released the first large-scale, expert-annotated dataset of key venous structures from pancreatic surgery (PS) videos and established benchmark performance for intraoperative vein segmentation using open-source models. This resource provides a foundation for advancing AI-assisted vascular identification in laparoscopic surgery.

## Introduction

The minimally invasive approach of laparoscopic surgery offers clear advantages over open procedures in the management of pancreatic diseases, including reduced surgical trauma and attenuated systemic reaction (1). However, the pancreas is surrounded by a dense network of arteries and veins; thus, tumor invasion into single or multiple blood vessels makes vascular manipulation a primary source of surgical risk (2, 3). While arterial anatomy, such as the gastroduodenal artery, is routinely emphasized (4), venous structures also require equal attention as they serve both as high-risk zones of bleeding and as indispensable anatomical landmarks. During both laparoscopic pancreaticoduodenectomy (LPD) and distal pancreatectomy (LDP), the superior mesenteric vein (SMV), portal vein (PV), and splenic vein (SV) require meticulous handling due to their thin, fragile walls and critical role as anatomical landmarks (5). Their exposure demands precision, as inadvertent injury to these structures may precipitate uncontrollable massive hemorrhage. The infiltration of the SMV-PV axis is one of the key indicators for assessing the surgical resectability of pancreatic cancer (6, 7), as well as tumors in the distal common bile duct, the duodenum and the area surrounding the ampulla of Vater. For resectable and borderline resectable pancreatic cancers, venous involvement often necessitates combined vein resection and reconstruction, substantially increasing surgical complexity (8–10). Improper vascular handling may result in severe postoperative complications, including pancreatic fistula, hemorrhage, gastroparesis, and portal vein thrombosis (11–13), underscoring the need for precise vascular management.

In laparoscopic pancreatic surgery (PS), visual identification of vessels is the sole means of anatomical recognition due to the lack of tactile feedback (14, 15). However, visual clarity is frequently compromised by intraoperative bleeding, smoke from electrocautery and cutting, and rapid camera movements, hindering accurate anatomical recognition (16, 17). Additionally, less-experienced surgeons may face elevated stress and reduced performance under such conditions (18, 19), further jeopardizing procedural safety and patient outcomes. These challenges underscore the urgent need for intraoperative assistance systems to enhance anatomical visualization and surgical precision.

Data-driven deep learning (DL) image recognition technology holds promise for mitigating challenges arising from the limitations of human vision and surgical inexperience. In diagnosis, DL systems such as the PANDA model have achieved expert-level performance in detecting subclinical pancreatic lesions via non-contrast abdominal CT scans (20). This model leverages a prospective cohort study of 3,000 subjects with standardized CT imaging, reflecting both significant scientific rigor and clinical value. For preoperative resectability assessment of PS, numerous researchers utilize imaging data for semantic segmentation and three-dimensional reconstruction to simulate anatomical relationships between pancreatic lesions and surrounding structures (21–23). This enables precise identification and localization of critical anatomy during surgery, providing visual decision support to enhance operative precision. However, as far as the surgical operation itself is concerned, there is an extreme lack of studies on intraoperative anatomical identification in PS. Beyond our team's publication of the first study on intraoperative vascular identification in pancreatic procedures (24), no other reports exist. This stands in remarkable contrast to the explosive growth in laparoscopic cholecystectomy (LC) research, where systematic breakthroughs have been achieved across multiple dimensions: anatomical structure recognition (25), surgical phase identification (26, 27), and critical view of safety (CVS) evaluation (28, 29). These advances are largely attributable to the release of Cholec80 (30), the largest sample size of annotated intraoperative LC image dataset at that time. Therefore, to accelerate intelligent research on PS, the creation of high-quality annotated intraoperative datasets has emerged as a critical bottleneck requiring urgent resolution.

To address this gap, we present VIP20K, the first expert-annotated dataset (20,000 images for Vein Identification in Pancreatic surgery) focused on key venous structures in laparoscopic PS. VIP20K concentrates on the need for real-time intraoperative identification of high-risk vessels such as the SMV-PV axis and the SV, and provides a foundation for advancing intraoperative vascular recognition. We further establish baseline semantic segmentation performance using models constructed entirely from publicly available components. This dataset is expected to facilitate the development of advanced algorithms for real-time, precise venous identification, ultimately reducing operative risk, lowering surgeon cognitive burden, and improving clinical outcomes in pancreatic surgery.

## Materials and methods

**Figure 1:**
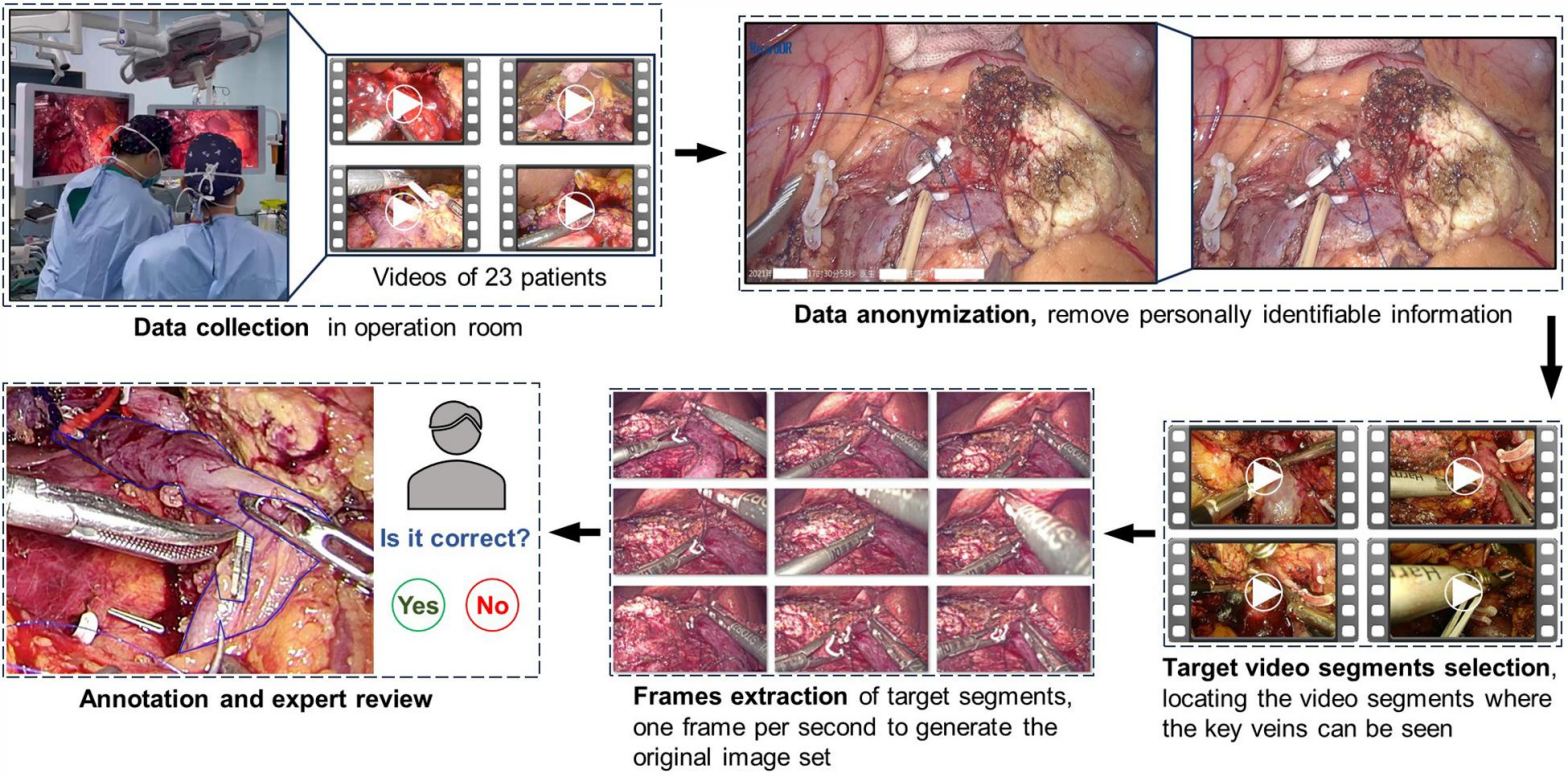
Overview of data collection process and methodology in the research.

Our dataset construction followed a five-stage workflow (Figure 1): data collection using standard laparoscopy equipment at Peking Union Medical College Hospital (PUMCH), data anonymization, target video segments selection, frames extraction, and key veins annotation and expert review. This methodical framework facilitates the creation of a standardized laparoscopic PS video repository with precise annotations and rigorous quality control.

### Surgical procedure

All operations were carried out in accordance with established minimally invasive techniques. The key steps are described as follows. In LPD, the gastrocolic ligament was divided, and the hepatic flexure was mobilized, followed by Kocher maneuver for duodeno-pancreatic mobilization. Vascular dissection of the hepatoduodenal ligament and gastroduodenal artery preceded inferior-border dissection of the pancreas, during which the SMV and PV became clearly visible. A tunnel was created between the pancreatic neck and SMV/PV before pancreatic-neck transection and completion of resection and reconstruction (31). In LDP, access to the lesser sac was achieved by dividing the gastrocolic ligament, followed by mobilization of the transverse colon and splenic flexure to expose the pancreatic body–tail. A tunnel was created between the pancreatic neck and the SMV and PV, and then the splenic vein and artery were dissected, and the pancreas was transected at the planned line using a linear stapler (32).

### Laparoscopic pancreatic surgery video acquisition

We retrospectively collected intraoperative videos from laparoscopic PS performed at PUMCH between January 2021 and June 2022. All procedures were performed by senior surgeons (each with >100 documented Whipple procedures) utilizing KANGJI® laparoscopic endoscopes capable of 4K imaging (3,840 × 2,160 pixels). Real-time recording was achieved via KJ.FPAK-02 recording hosts for intraoperative monitoring and archival. Video capture commenced upon placing laparoscopy into abdominal cavity through trocar and continued uninterrupted until removal of trocar symbolizing the end of the surgery, ensuring comprehensive procedural documentation. Video inclusion criteria: (a) Complete procedural record of LPD or LDP, (b) Continuous visualization of the SMV-PV axis for ≥2 min. Video exclusion criteria: (a) Intraoperative conversion to open surgery, (b) Prior abdominal or pelvic surgical interventions, (c) Confirmed vascular invasion or borderline resectable pancreatic cancer. After screening according to the standards, 23 surgeries were included, including 15 cases of LDP and 8 cases of LPD. The demographics and tumor details were illustrated in Table 1. All patient inclusion required both clinical indications for surgery and written informed consent. This study received ethical approval from the Ethics Committee of PUMCH (Approval NO. I-25PJ0451 for collection of surgical videos in the institution).

**Table 1:** Demographics and tumor characteristics of the patients.

ID	Age (years)	Gender	Surgical procedure	Tumor size on imaging (cm × cm)	Tumor location	Vascular invasion	Pathological diagnosis
LPD case 01	78	Female	LPD	3.0 × 4.5	Head of pancreas	No	Intraductal Papillary Mucinous Neoplasm of the Pancreas (IPMN)
LPD case 02	67	Male	LPD	1.0 × 1.1	Pancreatic segment of common bile duct	No	Adenocarcinoma of the Common Bile Duct
LPD case 03	67	Male	LPD	2.2 × 3.0	Ampulla of duodenum	No	Adenocarcinoma of the Duodenum
LPD case 04	63	Male	LPD	1.2 × 0.9	Pancreatic segment of common bile duct	No	Adenocarcinoma of the Common Bile Duct
LPD case 05	57	Male	LPD	1.8 × 1.5	Uncinate process of pancreas	No	Adenocarcinoma of the Pancreas
LPD case 06	69	Male	LPD	2.5 × 1.5	Ampullary segment of common bile duct	No	Adenocarcinoma of the Common Bile Duct
LPD case 07	38	Female	LPD	3.7 × 3.1	Head of pancreas	No	Solid Pseudopapillary Neoplasm of the Pancreas
LPD case 08	73	Female	LPD	2.9 × 2.0	Major duodenal papilla	No	Moderately Differentiated Adenocarcinoma of the Ampulla of Vater
LDP case 01	72	Male	LDP	4.2 × 4.7	Tail of pancreas	No	Adenocarcinoma of the Pancreas
LDP case 02	69	Male	LDP	3.0 × 2.8	Body of pancreas	No	Adenocarcinoma of the Pancreas
LDP case 03	58	Female	LDP	1.9 × 2.4	Body of pancreas	No	Adenocarcinoma of the Pancreas
LDP case 04	79	Male	LDP	4.3 × 5.2	Body of pancreas	No	Adenocarcinoma of the Pancreas
LDP case 05	64	Female	LDP	2.1 × 2.7	Body of pancreas	No	Borderline Tumor of the Pancreas
LDP case 06	65	Male	LDP	2.3 × 1.8	Body of pancreas	No	Adenocarcinoma of the Pancreas
LDP case 07	63	Female	LDP	2.4 × 3.1	Body of pancreas	No	Adenocarcinoma of the Pancreas
LDP case 08	73	Male	LDP	2.8 × 2.6	Body of pancreas	No	Adenocarcinoma of the Pancreas
LDP case 09	59	Female	LDP	2.5 × 3.3	Body of pancreas	No	Adenocarcinoma of the Pancreas
LDP case 10	77	Female	LDP	3.0 × 3.6	Body of pancreas	No	Adenocarcinoma of the Pancreas
LDP case 11	52	Male	LDP	1.4 × 2.2	Body of pancreas	No	Adenocarcinoma of the Pancreas
LDP case 12	61	Male	LDP	1.8 × 1.6	Body of pancreas	No	Adenocarcinoma of the Pancreas
LDP case 13	71	Female	LDP	2.1 × 1.9	Body of pancreas	No	Adenocarcinoma of the Pancreas
LDP case 14	65	Male	LDP	3.1 × 2.8	Body of pancreas	No	Adenocarcinoma of the Pancreas
LDP case 15	76	Female	LDP	2.3 × 2.9	Body of pancreas	No	Adenocarcinoma of the Pancreas

*LPD, laparoscopic pancreaticoduodenectomy; LDP, distal pancreatectomy; Vascular Invasion, assessing whether the mass invades critical blood vessels such as the celiac axis, portal vein, and superior mesenteric artery or vein.

### Data anonymization

Our core team members conducted comprehensive reviews of all surgical videos to identify and remove personally identifiable information of both physicians and patients. The processed videos were systematically renamed according to standardized protocols, and sidebars containing temporal data, device specifications, physician names, and patient medical record numbers were excised. Additionally, we deleted operationally irrelevant footage recorded when the laparoscope was temporarily withdrawn from the abdominal cavity for lens cleaning. These measures collectively ensured full data anonymization in accordance with general data protection regulation (GDPR) requirements.

### Target video segments selection and frames extraction

Senior surgeons systematically reviewed surgical videos to visually identify critical venous vasculature, precisely annotating frame-accurate start and end timestamps on the video timeline. Surgical scenes such as obvious bloodstains covering, smoke, and shaky camera movements were all included to enhance the richness of the video scenes. We then employed video processing algorithms to automatically extract all segments containing target anatomical structures based on this timestamp metadata, thus establishing an initial surgical video library for subsequent analysis and reference. Subsequently, frames were systematically extracted from these video segments at a fixed temporal interval of one frame per second, then stored as JPEG files with a resolution of 1,920 × 1,080 pixels to compile the source image dataset.

### Annotation and expert review

**Figure 2:**
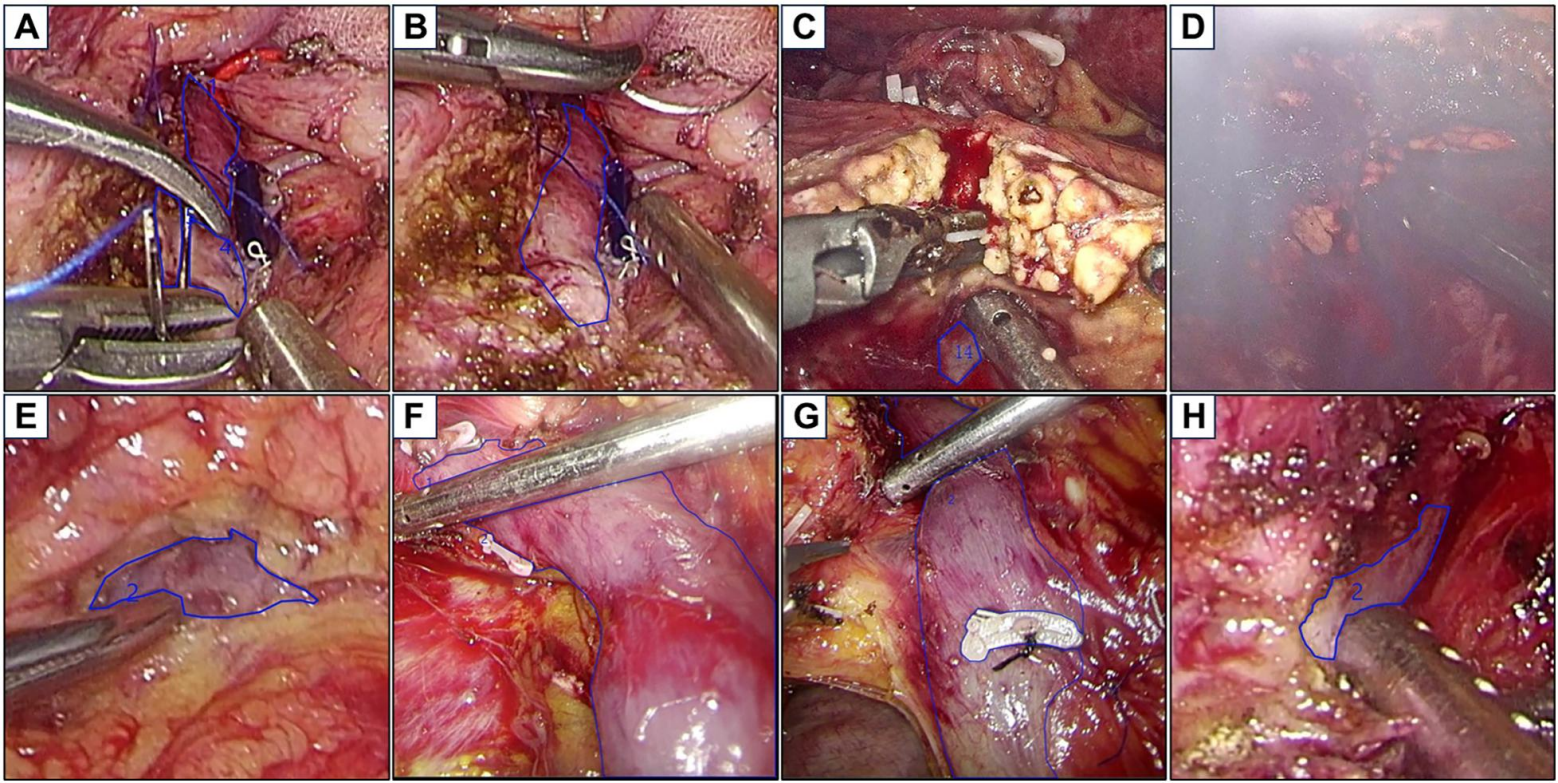
Examples of the critical vein annotations for different intraoperative scenarios. The blue polygon delineates the contour of the target vein. **(A)** Trace along the edge of the instruments during annotation, and bypass the obstructed area. **(B)** Disregard the suture and directly trace the contour of the vein beneath it. **(C)** Minimum annotation size requirement, exclude targets with minimum bounding rectangle ≤64 × 64 pixels. **(D)** Exclude images where the target veins are not clearly visible. **(E)** Outline the contours of the veins covered by thin fascial/adipose coverage. **(F)** Direct contour delineation of venous structures through thin blood layers, skip the instrument and mark each section separately. **(G)** Bypass superficial surgical instruments and thick fascial/adipose tissues that has not yet been stripped. **(H)** Annotations must exclude thick tissues and blood residues at marginal regions.

Following image dataset generation, four uniformly trained junior surgeons utilized our team's dedicated annotation software (supporting cross-device pixel-level annotation on tablets/desktops/laptops) to delineate contours of the SMV, PV, and SV based on standardized protocols. Key annotation criteria included (Figure 2): (a) Instrument obstruction handling: if the target vein is partially or completely obscured by surgical instruments, retraction cords, catheters, or other objects, trace along the edge of the obstruction during annotation, bypassing the obstructed area to reflect the actual visible portion of the vein; if the obstruction is a suture, disregard the suture and directly trace the contour of the vein beneath it. (b) Minimum annotation size requirement: target veins with area of minimum bounding rectangle box smaller than 64 × 64 pixels in the image are deemed ineligible for annotation and should be excluded. (c) Exclusion criteria for image quality: images exhibiting motion blur, fog interference, severe glare, or other artifacts affecting interpretation must be skipped during marking; images where the target vein is not within the field of view are also excluded. (d) Handling fascia and adipose tissue: direct contouring for thin fascial/adipose coverage over veins, whereas thick coverage required outlining along its periphery and bypassing the obscured area. (e) Blood interference processing: direct annotation for thin blood layers which permits venous wall visualization, exclusion for thick blood obscuring veins or obvious bloodstain at vessel margins. (f) Temporal consistency: for continuous frame sequences, annotations must maintain strict consistency across frames to prevent contour discrepancies of the same structure in different frames. All annotations underwent independent binary assessment (Yes/No) by two senior surgeons who completed over 100 Whipple procedures evaluating anatomical accuracy and protocol compliance. Annotations receiving unanimous approval were accepted; conflicting or rejected cases required re-annotation under senior guidance until consensus was achieved, developing a high-precision annotated image dataset.

### Technical validation

**Figure 3:**
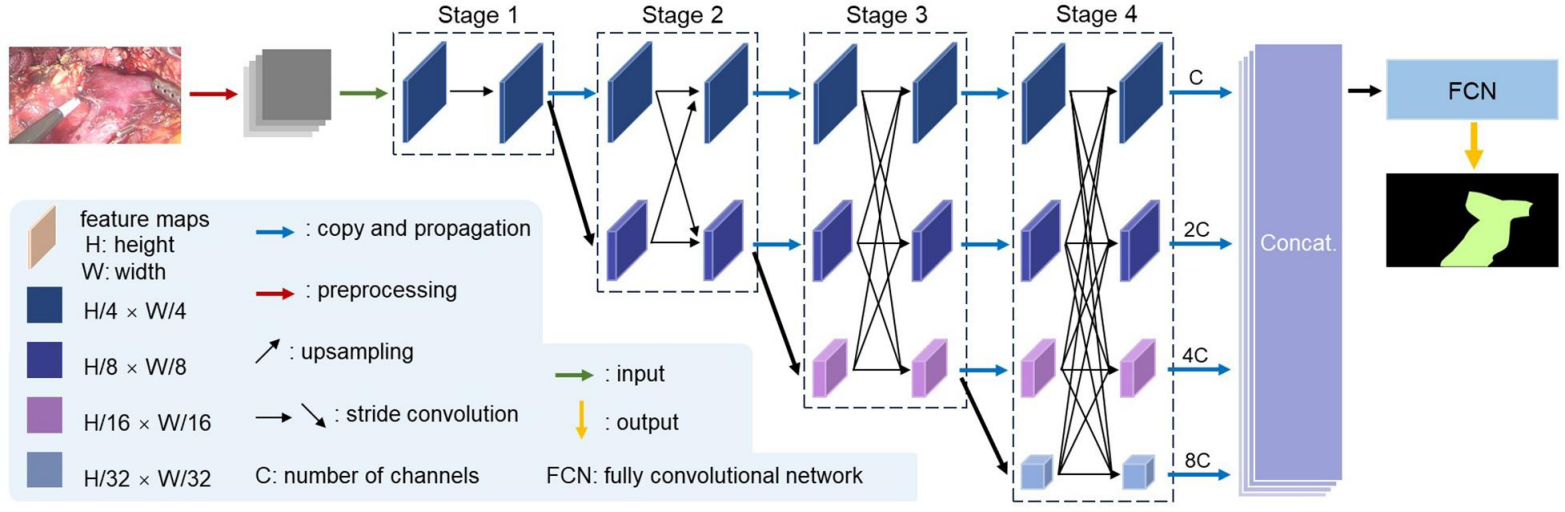
Overview of HRNet-FCN segmentation model.

In this semantic segmentation task targeting surgical scenarios, we employed High-Resolution Network (HRNet) as the backbone network, combined with a fully convolutional network (FCN) output head to construct the segmentation model (Figure 3). The adopted HRNet-W18-C variant is distinguished by its four-stage progressive multi-resolution architecture, which maintains high-resolution representations throughout the network (33). In the initial stage of the network, only the high-resolution branch was included. The original input image was convolved to a resolution of 1/4 scale and then input into HRNet. In this stage, the number of channels was 18, and residual convolution was used to extract basic spatial features. Subsequent stages progressively integrated low-resolution branches at 1/2, 1/4, and 1/8 resolutions, with channel counts doubling to 36, 72, and 144, respectively. Each branch utilized cross-resolution exchange units to enable feature interaction. High-resolution branches incorporated upsampled semantic information from low-resolution branches to enhance contextual awareness. Low-resolution branches integrated downsampled detail features from high-resolution branches to improve spatial sensitivity. This bidirectional information exchange, iteratively reinforced across residual units, established a hierarchical feature enhancement mechanism. By preserving high-resolution features and enabling dynamic multi-scale fusion, HRNet mitigated spatial information loss caused by repeated downsampling, particularly advantageous for precise localization of fine structures in surgical imaging such as vessels.

To adapt HRNet for semantic segmentation, we replaced its classification output head with an FCN output layer. The core principle of FCN lies in fully convolutional operations and end-to-end pixel-wise prediction (34). The FCN head employs deconvolution to refine segmentation boundaries, recovering spatial details lost during downsampling. The architecture we developed integrated high-resolution feature preservation with dynamic cross-scale fusion, leveraging FCN's pixel-level prediction capabilities to enable precise vein segmentation in complex surgical scenes.

Corresponding strategies were also taken in training configuration and optimization. The backbone weights of HRNet had been initialized through ImageNet pre-training to accelerate convergence. In data preprocessing, input images were resized to 960 × 540 while maintaining the original aspect ratio. From the resized images, 512 × 512 patches were randomly cropped and horizontally flipped with a 50% probability to increase spatial diversity. RGB channels were standardized with a mean of 123.68, 116.28, and 103.53, respectively, and a standard deviation of 57.12. No additional augmentation techniques were applied to preserve anatomical authenticity. The model was trained across 8 NVIDIA Tesla V100 GPUs with Python 3.6 and PyTorch 0.4.1. Training hyperparameters were as follows: a per-GPU batch size of 32, Stochastic Gradient Descent (SGD) Optimizer with momentum 0.9 and weight decay factor 0.0005, learning rate initialized at 0.01and decayed exponentially per epoch with a decay exponent of 0.9, and iterations for 100,000 epochs. To address severe pixel imbalance between the target veins and background, a weighted cross-entropy loss was employed:

$$L=-\frac{1}{n}\overset{n}{\underset{i=1}{\sum }}\;\underset{c\in C}{\sum }\;w_{c}\cdot y_{c,i}^{true}log(y_{c,i}^{\;pre})$$

$$w_{c}=\frac{total\;pixels\;n}{\;pixels\;in\;class\;c}=\frac{n}{n_{c}}$$

where *n* was total pixels per image, *i* was pixel index, *c* presented a class, $y_{c,i}^{true}$ was ground truth label, $y_{c,i}^{\;pre}$ was predicted probability, $w_{c}$ was weight of class c, and $n_{c}$ was the pixel count of class *c*. In postprocessing, morphological refinement was employed via cv2.morphologyEx to eliminate edge artifacts and connect fragmented regions.

For comprehensive evaluation of the segmentation model, we employed three key metrics to rigorously quantify performance: recall (sensitivity), precision, and Dice coefficient. Recall quantifies a model's detection completeness by measuring the ratio of true positives to all actual positives, emphasizing minimization of false negatives. Recall evaluates the proportion of among. Precision assesses prediction reliability through the fraction of true positives among all predicted positives, thereby reducing false alarms. Dice coefficient quantifies spatial overlap between predictions and ground truth, offering robustness against class imbalance.

$$Dice=\frac{2\;*\;area\;(Prediction)\;\cap \;area\;(Ground\;Truth)}{\sum area\;(Prediction)\;+\;\sum area\;(Ground\;Truth)}$$

## Results

### Dataset architecture

The complete dataset is publicly available on the Kaggle platform and can be accessed via https://www.kaggle.com/datasets/prohuge/vip20k. The root directory of the dataset, named VIP20K, comprises two subdirectories: LPD-PUMCH and LDP-PUMCH, which respectively store imaging data for two distinct pancreatic surgical procedures, laparoscopic pancreaticoduodenectomy and laparoscopic distal pancreatectomy. This segregated storage design facilitates cross-technique comparative studies, where LPD-PUMCH contains data from 8 LPD procedures (Case 01–08) and LDP-PUMCH includes 15 procedures (Case 01–15) (Table 2). Each case data is organized into a three-tiered hierarchical structure:

Case-level: Folders named by case identifiers (e.g., LPD-PUMCH/Case 01/) store comprehensive data for individual surgeries.
Segment-level: Subfolders are created under the case folder based on the surgical procedure sequence (e.g., LPD-PUMCH/Case 01/segment_01/), with each segment representing a continuous operational unit.
Data-level: Segment folders contain three subdirectories:
  3.1 Image/: Raw surgical scene images (JPEG format)
  3.2 Mask/: Pixel-wise annotation masks (PNG format)
  3.3 Merge/: Visual composite images (JPEG format) generated by overlaying masks on raw images.

**Table 2:** Size of the dataset.

Procedure type	Cases	Total segments	Image-mask pairs
Laparoscopic pancreaticoduodenectomy	8	44	10,737
Laparoscopic distal pancreatectomy	15	67	8,266
Total	23	111	19,003

The file naming convention ensures strict spatial consistency across modalities, for example, within the directory LPD-PUMCH/Case 01/segment_01, the files Image/00050.jpg, Mask/00050_mask.png, and Merge/00050.jpg represent the same frame. The resolution is uniformly set to 1,920 × 1,080 pixels to eliminate scale discrepancies. This dataset is organized using a standardized hierarchical structure, aiming to provide high-quality anatomical annotation resources for computer vision-assisted surgery research.

### Segmentation validation

**Figure 4:**
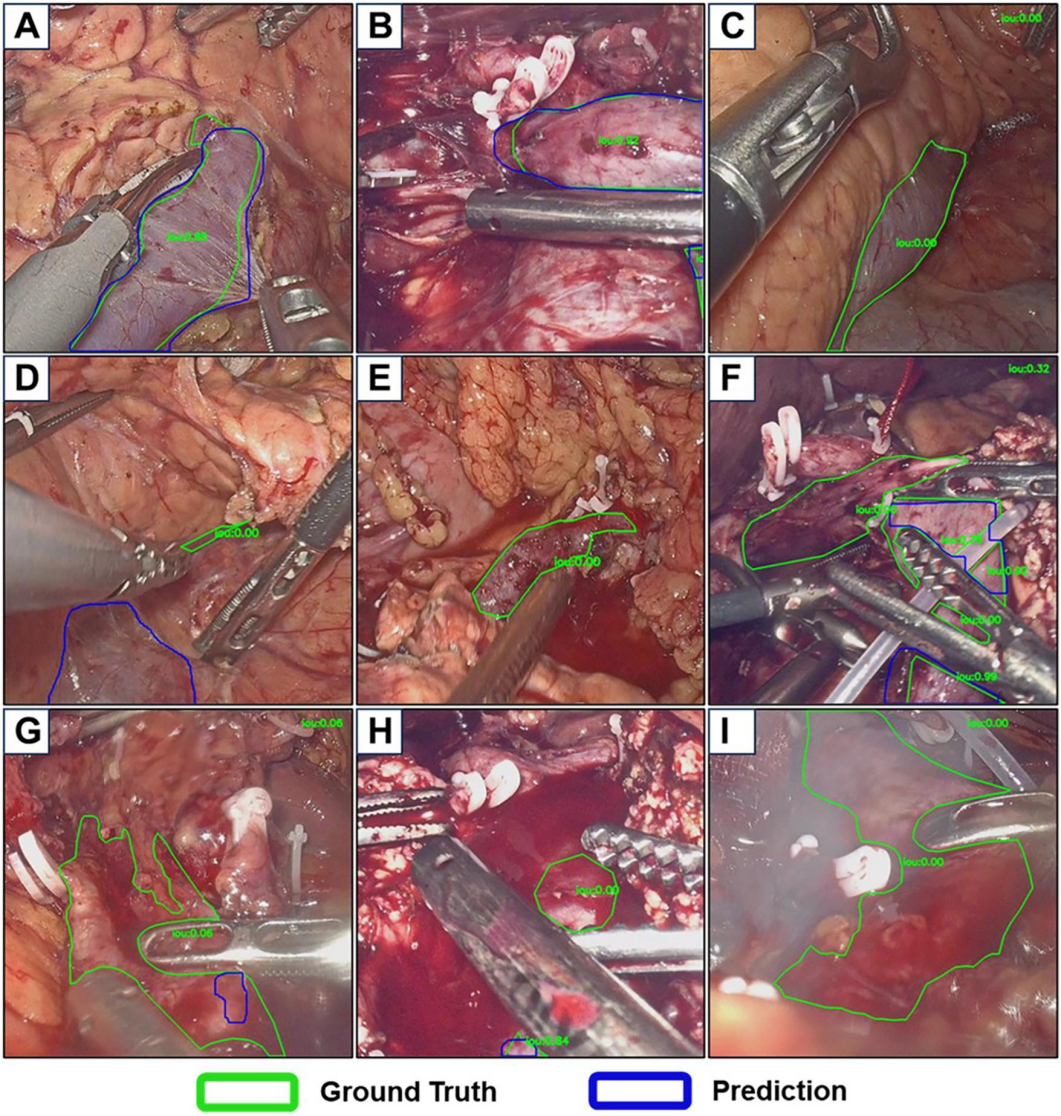
Examples of inference results from the semantic segmentation model. IoU: Intersection over Union, indicates the degree of overlap between the prediction bounding box and the ground truth bounding box. **(A)** Correct recognition of the splenic vein (SV) during laparoscopic distal pancreatectomy (LDP) surgery. **(B)** Correct recognition of the portal vein (PV) and superior mesenteric vein (SMV) during laparoscopic pancreaticoduodenectomy (LPD). **(C)** Failure to identify a visible SV during LDP. **(D)** Omission of a small-target SV with misidentification of other structures as the target vein in LDP. **(E)** Missed detection of the SV surrounded by blood during LDP. **(F)** Incomplete segmentation of the SMV-PV axis, with two regions unannotated in multi-area labeling, during LPD. **(G)** PV omission due to heavy surface blood coverage during LPD. **(H)** Failure to detect a PV segment submerged in pooled blood during LPD. **(I)** Missed SMV-PV identification in an obscured surgical view with smoke and blood interference during LPD.

In the validation of the HRNet-FCN model on the dataset, a 4:1 ratio was applied to partition the data into training and testing sets. To enhance the model's generalization capability across pancreatic surgical scenarios, data from two distinct procedures LDP and LPD were incorporated into the training phase. For the LDP cohort, 15 cases were included, with the training set comprising 6,417 frames from 14 patients and the testing set containing 1,849 frames from a single patient (Case 15). Similarly, in the LPD cohort with 8 cases, the training set consisted of 8,708 frames from 6 patients, while the testing set included 2,029 frames from 2 patients (Case 07 and Case 08). As summarized in Table 3, the model achieved a recall of 79.6%, precision of 95.8%, and Dice coefficient of 0.69 on the testing set, demonstrating robust practical utility. While false alarms were infrequent, false negatives or called miss rate particularly in veins detection warranted attention (Figure 4). Veins with a relatively large exposed area and clear surfaces were rarely missed, however, recognition failures occurred in scenes involving veins with small exposed area, blood occlusion, multiple segmented targets, or blurry scenes. These observations highlighted key areas for future optimization in venous segmentation under complex intraoperative conditions. The established performance metrics provide a comparative baseline for subsequent research in anatomical landmark identification during PS.

**Table 3:** Validation results of dataset.

Testing set	Frames	Recall	Precision	Miss rate	False alarm	Dice
LPD case 07	1,112	84.9%	91.8%	15.1%	8.2%	0.66
LPD case 08	917	84.2%	98.7%	15.8%	1.3%	0.76
LDP case 15	1,849	74.7%	96.5%	25.3%	3.5%	0.68
Total	3,878	79.6%	95.8%	20.4%	4.2%	0.69

## Discussion

This work introduced the first publicly available dataset specifically designed for intraoperative vascular recognition in PS, addressing a critical gap in surgical data science. Our dataset captures dynamic and diverse forms of the SMV, PV, and SV under realistic surgical conditions, including blood occlusion, instrument obstruction, and tissue deformation, which enabled model training for high-risk scenarios where vascular injury may lead to severe complications. By open-sourcing this resource, we encourage the community to engage in safety-critical computer vision applications.

The core value of this dataset stems from its complex pancreatic surgical scenes and annotation difficulty, necessitating close collaboration between surgeons and AI engineers to overcome intraoperative labeling challenges. Specifically, vessel visibility exhibits dynamic fluctuations due to interference factors such as bleeding, smoke interference, instrument movement, and changes in the surgical fields, requiring meticulous frame-by-frame review. Accurate delineation of vascular boundaries demands specialized surgical expertise to distinguish true vessel walls from artifacts, necessitating standardized training protocols for all annotators to ensure annotation consistency. The entire workflow, from video acquisition, annotation, to quality review, and dataset finalization, involves over 1,000 h of work, highlighting the tremendous effort required to develop a high-precision annotated surgical vision dataset.

The HRNet-FCN architecture demonstrates exceptional performance in vessel segmentation by leveraging its intrinsic high-resolution feature preservation mechanism. Through parallel multi-scale branches, HRNet maintains spatial accuracy at 1/4 input resolution, significantly mitigating information loss typically observed in encoder-decoder architectures. The model's robustness is further evidenced by its Dice coefficient of 0.69 in laparoscopic PS, enabling precise anatomical landmark identification while reducing risk of critical vein injury. We assembled a straightforward baseline model from open-source components; its performance on the proposed dataset can serve as a reference for other researchers to innovate in architecture design and algorithmic development, thereby advancing intraoperative vein recognition accuracy and ultimately enhancing clinical benefit for surgeons. This initiative aims to enhance intraoperative vein recognition accuracy, ultimately advancing clinical benefits for surgeons and patients through improved surgical safety and decision support.

Limitations remain that must guide future work in the research. The current dataset exhibits several limitations. First, the limited sample size of 23 patients, while consistent with the pilot nature of this work, may constrain the generalizability of the model to the broader population of patients undergoing PS. Second, all data were obtained from a single medical center. Although the use of standardized instruments and procedures ensures internal consistency, it may limit the model's applicability across diverse clinical settings and potentially introduce systematic biases. Third, while the dataset is the largest publicly available resource (20,000 frames) for identifying important veins in PS, the incidence of clinically pivotal scenarios, including massive hemorrhage and vascular anomalies, remains low. Thus, the model's robustness in identifying the rare but high-stakes events is constrained. Furthermore, reliance solely on intraoperative video neglects the guidance value of preoperative planning. This creates a fragmented decision-making workflow, requiring surgeons to manually cross-reference preoperative CT/MR vascular reconstructions across isolated systems, which compromises operational efficiency and spatial accuracy. Regarding model performance, the current framework demonstrates suboptimal detection sensitivity for veins with limited exposure and in blood-occluded scenarios. Enhancing recognition under these challenging conditions necessitates innovations in both deep learning architectures and multimodal fusion strategies.

In the future, there are some directions that can be explored. To enhance model generalizability, a cross-institutional federated learning system will integrate multi-center data using encrypted parameter exchange and feature alignment techniques, ensuring privacy-compliant collaboration across hospitals. Generative adversarial networks (GANs) will synthesize high-fidelity intraoperative hemorrhage and vascular anomaly samples to address class imbalance in rare scenarios. For operating-room deployment, neural architecture search (NAS) and quantization-aware training (QAT) will optimize models for embedded systems. A transformer-based cross-modal registration mechanism will align intraoperative video with preoperative CT/MR vascular models at a precision of millimeter level, adapting dynamically to tissue deformation. This enables an augmented reality navigation platform that overlays real-time segmentation and surgical pathways onto the surgical field, providing millimeter level guidance accuracy.

## Conclusion

This study constructed and released the first large-scale annotated dataset of major veins sourced from PS videos. We validate its usability through baseline evaluations using open-source models, establishing benchmark metrics for intraoperative vein segmentation. This resource will empower practitioners to innovate algorithms and models, advancing the precision and efficiency of venous structure identification during surgery, ultimately translating into direct clinical benefits for patients and surgeons.

## Data Availability

The datasets presented in this study can be found in online repositories. The names of the repository/repositories and accession number(s) can be found below: www.kaggle.com/datasets/prohuge/vip20k.

## References

[1] Tan-Tam C Chung SW. Minireview on laparoscopic hepatobiliary and pancreatic surgery. World J Gastrointest Endosc. (2014) 6(3):60–7. 10.4253/wjge.v6.i3.60 24634709 PMC3952161

[2] Filipoiu FM Badea GT Enyedi M Oprea Ș Filipoiu ZF Mutu DEG. Mesopancreas-anatomical insights and its implications for diagnosis and clinical and surgical practice. Diagnostics (Basel). (2025) 15(7):20–1. 10.3390/diagnostics15070914 PMC11989011 40218264

[3] Tseng JF Raut CP Lee JE Pisters PWT Vauthey JN Abdalla EK Pancreaticoduodenectomy with vascular resection: margin status and survival duration. J Gastrointest Surg. (2004) 8(8):935–49. discussion 949–950. 10.1016/j.gassur.2004.09.046 15585381

[4] Yamaguchi T Hasegawa K Sauvain MO Passoni S Kazami Y Kokudo T An aberrant right hepatic artery arising from the gastroduodenal artery: a pitfall encountered during pancreaticoduodenectomy. Surg Today. (2021) 51(10):1577–82. 10.1007/s00595-021-02242-4 33575949

[5] Ball CG Dixon E Vollmer CM Howard TJ. The view from 10,000 procedures: technical tips and wisdom from master pancreatic surgeons to avoid hemorrhage during pancreaticoduodenectomy. BMC Surg. (2015) 15:122. 10.1186/s12893-015-0109-y 26608343 PMC4660662

[6] Xu D Wu P Zhang K Cai B Yin J Shi G The short-term outcomes of distal pancreatectomy with portal vein/superior mesenteric vein resection. Langenbecks Arch Surg. (2022) 407(5):2161–8. 10.1007/s00423-021-02382-8 35606575

[7] Lopez NE Prendergast C Lowy AM. Borderline resectable pancreatic cancer: definitions and management. World J Gastroenterol. (2014) 20(31):10740–51. 10.3748/wjg.v20.i31.10740 25152577 PMC4138454

[8] Javed AA Bleich K Bagante F He J Weiss MJ Wolfgang CL Pancreaticoduodenectomy with venous resection and reconstruction: current surgical techniques and associated postoperative imaging findings. Abdom Radiol (NY). (2018) 43(5):1193–203. 10.1007/s00261-017-1290-5 28828527

[9] Ramacciato G Mercantini P Petrucciani N Giaccaglia V Nigri G Ravaioli M Does portal-superior mesenteric vein invasion still indicate irresectability for pancreatic carcinoma? Ann Surg Oncol. (2009) 16(4):817–25. 10.1245/s10434-008-0281-8 19156463

[10] Giovinazzo F Turri G Katz MH Heaton N Ahmed I. Meta-analysis of benefits of portal-superior mesenteric vein resection in pancreatic resection for ductal adenocarcinoma. Br J Surg. (2016) 103(3):179–91. 10.1002/bjs.9969 26663252

[11] Hackert T Klaiber U Hinz U Strunk S Loos M Strobel O Portal vein resection in pancreatic cancer surgery: risk of thrombosis and radicality determine survival. Ann Surg. (2023) 277(6):e1291–8. 10.1097/SLA.0000000000005444 35793384

[12] Lu JW Ding HF Wu XN Liu XM Wang B Wu Z Intra-abdominal hemorrhage following 739 consecutive pancreaticoduodenectomy: risk factors and treatments. J Gastroenterol Hepatol. (2019) 34(6):1100–7. 10.1111/jgh.14560 30511762

[13] Kokkinakis S Kritsotakis EI Maliotis N Karageorgiou I Chrysos E Lasithiotakis K. Complications of modern pancreaticoduodenectomy: a systematic review and meta-analysis. Hepatobiliary Pancreat Dis Int. (2022) 21(6):527–37. 10.1016/j.hbpd.2022.04.006 35513962

[14] Othman W Vandyck KE Abril C Barajas-Gamboa JS Pantoja JP Kroh M Stiffness assessment and lump detection in minimally invasive surgery using in-house developed smart laparoscopic forceps. IEEE J Transl Eng Health Med. (2022) 10:2500410. 10.1109/JTEHM.2022.3180937 35774413 PMC9216325

[15] Al-Taan OS Stephenson JA Briggs C Pollard C Metcalfe MS Dennison AR. Laparoscopic pancreatic surgery: a review of present results and future prospects. HPB (Oxford). (2010) 12(4):239–43. 10.1111/j.1477-2574.2010.00168.x 20590893 PMC2873646

[16] Lin X Fan Q Li R Chen R Yang Z Li Y. Enhancing laparoscopic visibility: efficient surgical smoke clearance innovatively using nebulization technology. Biomed Eng Online. (2025) 24(1):65. 10.1186/s12938-025-01395-4 40420139 PMC12105325

[17] Nwoye CI Mutter D Marescaux J Padoy N. Weakly supervised convolutional LSTM approach for tool tracking in laparoscopic videos. Int J Comput Assist Radiol Surg. (2019) 14(6):1059–67. 10.1007/s11548-019-01958-6 30968356

[18] Stoop TF Ateeb Z Ghorbani P Scholten L Arnelo U Besselink MG Surgical outcomes after total pancreatectomy: a high-volume center experience. Ann Surg Oncol. (2021) 28(3):1543–51. 10.1245/s10434-020-08957-x 32761326

[19] Wang M Peng B Liu J Yin X Tan Z Liu R Practice patterns and perioperative outcomes of laparoscopic pancreaticoduodenectomy in China: a retrospective multicenter analysis of 1029 patients. Ann Surg. (2021) 273(1):145–53. 10.1097/SLA.0000000000003190 30672792

[20] Cao K Xia Y Yao J Han X Lambert L Zhang T Large-scale pancreatic cancer detection via non-contrast CT and deep learning. Nat Med. (2023) 29(12):3033–43. 10.1038/s41591-023-02640-w 37985692 PMC10719100

[21] Miyamoto R Takahashi A Ogasawara A Ogura T Kitamura K Ishida H Three-dimensional simulation of the pancreatic parenchyma, pancreatic duct and vascular arrangement in pancreatic surgery using a deep learning algorithm. PLoS One. (2022) 17(10):e0276600. 10.1371/journal.pone.0276600 36306322 PMC9616217

[22] Akbari H Kosugi Y Khorgami Z. Image-guided preparation of the calot’s triangle in laparoscopic cholecystectomy. Annu Int Conf IEEE Eng Med Biol Soc. (2009) 2009:5649–52. 10.1109/IEMBS.2009.5333766 19964407

[23] Lin C Gao J Zheng H Zhao J Yang H Lin G Three-dimensional visualization technology used in pancreatic surgery: a valuable tool for surgical trainees. J Gastrointest Surg. (2020) 24(4):866–73. 10.1007/s11605-019-04214-z 31012044 PMC7165138

[24] Shi J Cui R Wang Z Yan Q Ping L Zhou H Deep learning HRNet FCN for blood vessel identification in laparoscopic pancreatic surgery. NPJ Digit Med. (2025) 8(1):235. 10.1038/s41746-025-01663-6 40312536 PMC12046043

[25] Silva B Oliveira B Morais P Buschle LR Correia-Pinto J Lima E Analysis of current deep learning networks for semantic segmentation of anatomical structures in laparoscopic surgery. Annu Int Conf IEEE Eng Med Biol Soc. (2022) 2022:3502–5. 10.1109/EMBC48229.2022.9871583 36085761

[26] Guédon ACP Meij SEP Osman KNMMH Kloosterman HA van Stralen KJ Grimbergen MCM Deep learning for surgical phase recognition using endoscopic videos. Surg Endosc. (2021) 35(11):6150–7. 10.1007/s00464-020-08110-5 33237461

[27] Shinozuka K Turuda S Fujinaga A Nakanuma H Kawamura M Matsunobu Y Artificial intelligence software available for medical devices: surgical phase recognition in laparoscopic cholecystectomy. Surg Endosc. (2022) 36(10):7444–52. 10.1007/s00464-022-09160-7 35266049 PMC9485170

[28] Mascagni P Vardazaryan A Alapatt D Urade T Emre T Fiorillo C Artificial intelligence for surgical safety: automatic assessment of the critical view of safety in laparoscopic cholecystectomy using deep learning. Ann Surg. (2022) 275(5):955–61. 10.1097/SLA.0000000000004351 33201104

[29] Wu S Tang M Liu J Qin D Wang Y Zhai S Impact of an AI-based laparoscopic cholecystectomy coaching program on the surgical performance: a randomized controlled trial. Int J Surg. (2024) 110(12):7816–23. 10.1097/JS9.0000000000001798 38896869 PMC11634122

[30] Twinanda AP Shehata S Mutter D Marescaux J de Mathelin M Padoy N. Endonet: a deep architecture for recognition tasks on laparoscopic videos. IEEE Trans Med Imaging. (2017) 36(1):86–97. 10.1109/TMI.2016.2593957 27455522

[31] Tuan LQA Hai PM. Laparoscopic pancreaticoduodenectomy. In: Lomanto D Tzu-Liang Chen W Fuentes MB, editors. Mastering Endo-Laparoscopic and Thoracoscopic Surgery: ELSA Manual. Singapore: Springer (2022). p. 357–66.

[32] Hai PM Tuan LQA. Laparoscopic distal pancreatectomy. In: Lomanto D Tzu-Liang Chen W Fuentes MB, editors. Mastering Endo-Laparoscopic and Thoracoscopic Surgery: ELSA Manual. Singapore: Springer (2022). p. 349–55.

[33] Wang J Sun K Cheng T Jiang B Deng C Zhao Y Deep high-resolution representation learning for visual recognition. IEEE Trans Pattern Anal Mach Intell. (2021) 43(10):3349–64. 10.1109/TPAMI.2020.2983686 32248092

[34] Long J Shelhamer E Darrell T. Fully convolutional networks for semantic segmentation. Proceedings of the IEEE Conference on Computer Vision and Pattern Recognition (2015). p. 3431–40 10.1109/TPAMI.2016.2572683 27244717

